# Supplementary material for: Synergistic effects of virtual reality and traditional treatment methods in the management of chronic obstructive pulmonary disease: a systematic review and meta-analysis of randomized controlled trials
Source: PeerJ. 2025 Oct 30;13:e20047. doi: 10.7717/peerj.20047 (PMC12579849; doi:10.7717/peerj.20047)
Supplement: Supplemental Information 1 [file peerj-13-20047-s001.docx]

pubmed:

(((Chronic Obstructive Pulmonary Disease[Title/Abstract]) OR (COPD[Title/Abstract])) AND ((Virtual Reality) OR (VR))) AND ((((((Drug Therapy[Title/Abstract]) OR (Pharmacotherapy[Title/Abstract])) OR (Medications[Title/Abstract])) OR (Physical Therapy[Title/Abstract])) OR (Oxygen Therapy[Title/Abstract])) OR (Pulmonary Rehabilitation[Title/Abstract]))
