# Supplementary material for: Synergistic effects of virtual reality and traditional treatment methods in the management of chronic obstructive pulmonary disease: a systematic review and meta-analysis of randomized controlled trials
Source: PeerJ. 2025 Oct 30;13:e20047. doi: 10.7717/peerj.20047 (PMC12579849; doi:10.7717/peerj.20047)
Supplement: Supplemental Information 3 [file peerj-13-20047-s003.docx]

The intended audience for this meta-analysis includes healthcare professionals, including clinicians and respiratory therapists, who are involved in the management of Chronic Obstructive Pulmonary Disease (COPD). Additionally, researchers interested in the integration of emerging technologies, such as virtual reality (VR), in traditional treatment modalities will find this work valuable. The study may also appeal to healthcare policymakers and educators who are exploring innovative treatment methods to improve patient outcomes and rehabilitation strategies. Furthermore, this research targets academic audiences, particularly those in the fields of pulmonology, rehabilitation medicine, and health technology, as well as multidisciplinary teams involved in the treatment and management of chronic diseases.
